# The association between hearing loss in adults and cognitive outcomes: an umbrella review and meta-analysis

**DOI:** 10.3389/fnagi.2026.1852163

**Published:** 2026-08-24

**Authors:** Jiao Liang, Penglong Yu, Qingzhu You, Kun Yang

**Affiliations:** Chongqing Three Gorges Medical College, College of Traditional Chinese Medicine, Chongqing, China

**Keywords:** Alzheimer’s disease, cognitive impairment, dementia, hearing loss, meta-analysis, umbrella review

## Abstract

**Purpose:**

Hearing loss (HL) has been increasingly recognized as a potential risk factor for cognitive decline and dementia. However, findings from existing meta-analyses remain heterogeneous. This umbrella review aimed to comprehensively synthesize current meta-analytic evidence on the association between adult hearing loss and cognitive outcomes.

**Methods:**

PubMed, Embase, Web of Science, and the Cochrane Library were systematically searched from inception to 9 January 2026. Systematic reviews with meta-analyses examining the relationship between hearing loss and cognitive outcomes in adults were included. Methodological quality was assessed using AMSTAR-2. Evidence credibility was evaluated using the Ioannidis classification framework, and certainty of evidence was assessed using the GRADE approach. Overlap among primary studies across meta-analyses was quantified using the corrected covered area (CCA).

**Results:**

Nine meta-analyses were included. Hearing loss was significantly associated with increased risk of cognitive impairment (OR = 1.34, 95% CI: 1.17–1.53) and dementia (OR = 1.43, 95% CI: 1.21–1.68). In older adults, the associations were slightly stronger. Hearing loss was also associated with higher risk of Alzheimer’s disease (OR = 1.66, 95% CI: 1.33–2.07). Evidence credibility was classified as highly suggestive for cognitive impairment and dementia.

**Conclusion:**

Adult hearing loss is significantly associated with increased risks of cognitive impairment and dementia. These findings highlight the importance of hearing health in cognitive aging and support further prospective and interventional studies.

## 1 Introduction

Hearing loss (HL) is one of the most common sensory impairments worldwide and poses a major challenge to public health and social wellbeing (Nieman and Oh, 2020). With the ongoing acceleration of global population aging, the prevalence of hearing loss continues to rise, particularly among middle-aged and older adults. According to the World Health Organization (WHO), more than 1.5 billion people worldwide are affected by varying degrees of hearing loss, a substantial proportion of whom are older adults (Chadha et al., 2021). Hearing loss not only impairs verbal communication, but may also reduce social participation, diminish quality of life, and contribute to a range of mental health problems, including depression and loneliness (Shukla et al., 2020). Therefore, hearing loss has been recognized as an important public health issue affecting health status and functional independence in older adults.

In recent years, increasing attention has been paid to the potential association between hearing loss and cognitive function. A growing body of evidence suggests that hearing loss may be associated with an increased risk of cognitive decline, mild cognitive impairment (MCI), and dementia (An et al., 2023; Chern and Golub, 2019; Lin et al., 2011; Slade et al., 2020). Several hypotheses have been proposed to explain this relationship. For example, the sensory deprivation hypothesis suggests that long-term reduction in auditory input may lead to structural and functional degeneration in related brain regions, thereby impairing cognitive function (Wayne and Johnsrude, 2015). The cognitive load hypothesis proposes that hearing loss increases the cognitive resources required for speech comprehension, thereby reducing the resources available for other cognitive tasks (Pichora-Fuller et al., 2016). In addition, hearing loss may indirectly affect cognitive function by promoting social isolation, reducing social interaction, and accelerating neurodegenerative changes. These mechanisms provide an important theoretical basis for understanding the association between hearing loss and cognitive decline.

Notably, hearing loss has been identified as one of the most important potentially modifiable risk factors for dementia (Livingston et al., 2020). Although a large number of observational studies have examined the relationship between hearing loss and cognitive outcomes, several systematic reviews and meta-analyses have also been published in recent years, with inconsistent findings across studies (Fu et al., 2022; Jiang et al., 2024; Loughrey et al., 2018). On the one hand, these meta-analyses differ in the number of included studies, study designs, and participant characteristics. On the other hand, definitions and measurement methods for both hearing loss and cognitive outcomes are not entirely consistent across studies. Moreover, most existing meta-analyses have primarily focused on pooled effect estimates, while relatively few have systematically assessed the overall reliability of the evidence, risk of bias, and certainty of evidence (Ioannidis, 2016). Therefore, the current body of evidence still lacks a higher-level, systematic synthesis and evaluation.

An umbrella review is a research approach that re-synthesizes findings from existing systematic reviews and meta-analyses, allowing a higher-level assessment of the consistency, reliability, and strength of evidence across studies (Aromataris et al., 2015). Although a previous umbrella review (Ying et al., 2023) has summarized the association between age-related hearing loss, cognitive impairment, and dementia, important evidence gaps remain. That review primarily focused on age-related hearing loss in older adults and included evidence available up to February 2023. Since then, additional systematic reviews and meta-analyses have been published, including studies addressing adult-onset hearing loss, longitudinal cognitive outcomes, and multiple cognitive domains. Moreover, the independence and credibility of the accumulated evidence require further evaluation because overlapping primary studies may influence the interpretation of umbrella-review findings. Therefore, the present umbrella review aimed to provide an updated and broader synthesis of meta-analytic evidence on the association between hearing loss in adults and cognitive outcomes. In addition to estimating associations with cognitive impairment, dementia, and Alzheimer’s disease, we further summarized cognitive-domain findings where available, assessed overlap among primary studies using the corrected covered area, and evaluated the credibility and certainty of evidence using established umbrella-review and GRADE frameworks.

## 2 Methods

### 2.1 Study design and protocol registration

This study was conducted as an umbrella review that systematically included and synthesized previously published systematic reviews and meta-analyses to evaluate the strength of evidence for the association between hearing loss and cognitive outcomes in adults. The design and reporting of this study followed the PRISMA 2020 statement (Page et al., 2021), and evidence credibility was classified according to the recommended methodological framework for umbrella reviews (Fusar-Poli and Radua, 2018). The protocol of this umbrella review was prospectively registered in the PROSPERO database (registration number: CRD420261355918).

### 2.2 Literature search

The PubMed, Embase, Web of Science, and Cochrane Library databases were systematically searched for relevant studies from inception to 9 January 2026. In addition, the reference lists of included studies were manually screened to identify potentially relevant articles that may have been missed in the database search. The search strategy consisted of two main groups of keywords: hearing loss–related terms (e.g., hearing loss, hearing impairment, deafness) and cognition-related terms (e.g., cognition, cognitive decline, cognitive impairment, mild cognitive impairment, dementia, and Alzheimer’s disease). In PubMed and the Cochrane Library, both MeSH terms and free-text terms were used, whereas in Embase, Emtree terms were combined with free-text terms. Truncation symbols and Boolean operators (“AND” and “OR”) were applied to construct the search strategy. The detailed search strategy is provided in the Supplementary Data Sheet 1.

### 2.3 Eligibility criteria

#### 2.3.1 Inclusion criteria

Studies were included if they met the following criteria:

(1) Study design: systematic reviews with meta-analysis, published in English;
(2) Participants: adults aged ≥ 18 years;
(3) Exposure: hearing loss or hearing impairment, assessed using self-report, pure-tone audiometry, or other objective or subjective methods;
(4) Comparator: individuals with normal hearing or without hearing loss;
(5) Outcomes: cognitive-related outcomes, including cognitive impairment, dementia risk, Alzheimer’s disease, longitudinal cognitive decline, or cognitive function;
(6) Effect estimates: studies reporting extractable effect measures [e.g., odds ratios (OR), risk ratios (RR), hazard ratios (HR), or standardized mean differences (SMD)] with corresponding 95% confidence intervals (CIs), or providing sufficient data for calculation.

#### 2.3.2 Exclusion criteria

Studies were excluded if they met any of the following criteria:

(1) Narrative reviews, expert opinions, editorials, conference abstracts, or study protocols without meta-analytic results;
(2) Studies including only children or adolescents, or those in which adult data could not be extracted separately;
(3) Studies that did not report cognitive-related outcomes (e.g., cognitive impairment or dementia);
(4) Duplicate publications or meta-analyses with substantial overlap in included studies. In such cases, priority was given to the most recent review with the most comprehensive inclusion of studies and higher methodological quality. If different meta-analyses focused on distinct outcome indicators, they were included separately and the overlap was addressed in the overlap assessment.

### 2.4 Study selection

All retrieved records were imported into EndNote X9 (Clarivate Analytics) for duplicate removal. Subsequently, two reviewers independently conducted a two-stage screening process: (Nieman and Oh, 2020) initial screening based on titles and abstracts, and (Chadha et al., 2021) full-text assessment for eligibility. Any disagreements were resolved through discussion, and if necessary, a third reviewer was consulted to reach a final decision. The study selection process was reported using a PRISMA flow diagram, with the primary reasons for exclusion documented.

### 2.5 Data extraction

Data were independently extracted by two reviewers and cross-checked for accuracy. Any discrepancies were resolved through discussion or consultation with a third reviewer. Extracted information included, but was not limited to: basic study characteristics (first author, publication year, and country/region); characteristics of the meta-analyses (number of included primary studies and total sample size); exposure definition (methods used to assess hearing loss, including pure-tone audiometry thresholds, self-reported measures, speech audiometry, central auditory tests, or medical records/diagnostic codes, and severity classification if available); outcome information (types of cognitive-related outcomes); and statistical information (type of pooled effect estimates, including OR, RR, HR, or SMD; effect sizes and corresponding 95% confidence intervals; heterogeneity indicators such as I^2^; number of included primary studies and sample size; and direction of the association). If a meta-analysis reported multiple outcomes or results at different time points, these data were extracted separately and organized according to outcome categories.

### 2.6 Quality assessment

The methodological quality of the included systematic reviews was evaluated using the AMSTAR-2 tool, which consists of 16 items, including seven critical domains and nine non-critical domains. Two reviewers independently conducted the quality assessment, and any disagreements were resolved through consultation with a third reviewer. According to the AMSTAR-2 recommended criteria, studies were categorized as high quality (no critical flaws and ≤1 non-critical weakness), moderate quality (one critical flaw and ≤1 non-critical weakness), low quality (>1 critical flaw and ≤1 non-critical weakness), or critically low quality (multiple critical flaws or a critical flaw combined with ≥2 non-critical weaknesses) (Shea et al., 2017).

### 2.7 Statistical analysis

Pooled effect estimates and their corresponding 95% confidence intervals (CIs) were extracted from each included systematic review. For studies reporting risk ratios (RR), hazard ratios (HR), or odds ratios (OR), ORs were used as the common effect measure to improve comparability across studies and were used for the re-meta-analysis (Liu et al., 2024; Zhang and Yu, 1998). For continuous cognitive outcomes, standardized mean differences (SMDs) were used. Statistical significance was set at *P* < 0.05. The choice of pooling model was based on the level of heterogeneity. A fixed-effect model was applied when I^2^ < 50%, whereas a random-effects model was used when I^2^ ≥ 50%. Statistical heterogeneity among studies was assessed using the I^2^ statistic. Considering the strong age dependence of the association between hearing loss and cognitive outcomes, subgroup analyses focusing on older adults were conducted in addition to analyses of the overall adult population. The older adult subgroup was defined as studies in which the majority of participants were older individuals, typically with age distributions concentrated at ≥55–60 years or explicitly described as older adults in the original reviews. To evaluate the robustness of the results, leave-one-out sensitivity analyses were performed for outcomes that included ≥5 studies. For meta-analyses including ≥10 studies, potential publication bias was assessed using Egger’s regression test. An Egger’s test *P*-value < 0.05 indicated possible small-study effects. When potential publication bias was detected, the trim-and-fill method was applied for further adjustment. To assess the overlap of primary studies across the included systematic reviews, the corrected covered area (CCA) method was used to construct a study-citation matrix and quantify the degree of overlap (Hennessy and Johnson, 2019). According to the criteria proposed by Pieper et al. (2014), overlap was categorized as slight (0%–5%), moderate (6%–10%), high (11%–15%), or very high (>15%). In cases of substantial overlap, priority was given to reviews with higher AMSTAR scores, larger sample sizes, or broader coverage of primary studies. All statistical analyses were performed using R software (version 4.5.0).

### 2.8 Level of evidence

For each outcome, the credibility of the evidence was classified according to commonly used umbrella review criteria proposed by Bellou et al. (2015). Evidence was categorized into four levels: Class I (convincing evidence), Class II (highly suggestive evidence), Class III (suggestive evidence), and Class IV (weak evidence). This classification was based on several criteria, including statistical significance, sample size, significance of the largest study, heterogeneity, prediction intervals, and potential bias. In addition, the certainty of evidence for each included meta-analysis was evaluated using the GRADE approach (Balshem et al., 2011). The GRADE framework assesses evidence quality across five domains: risk of bias, inconsistency, indirectness, imprecision, and publication bias. Based on these domains, the overall certainty of evidence was categorized as high, moderate, low, or very low.

## 3 Results

### 3.1 Study selection

**FIGURE 1:**
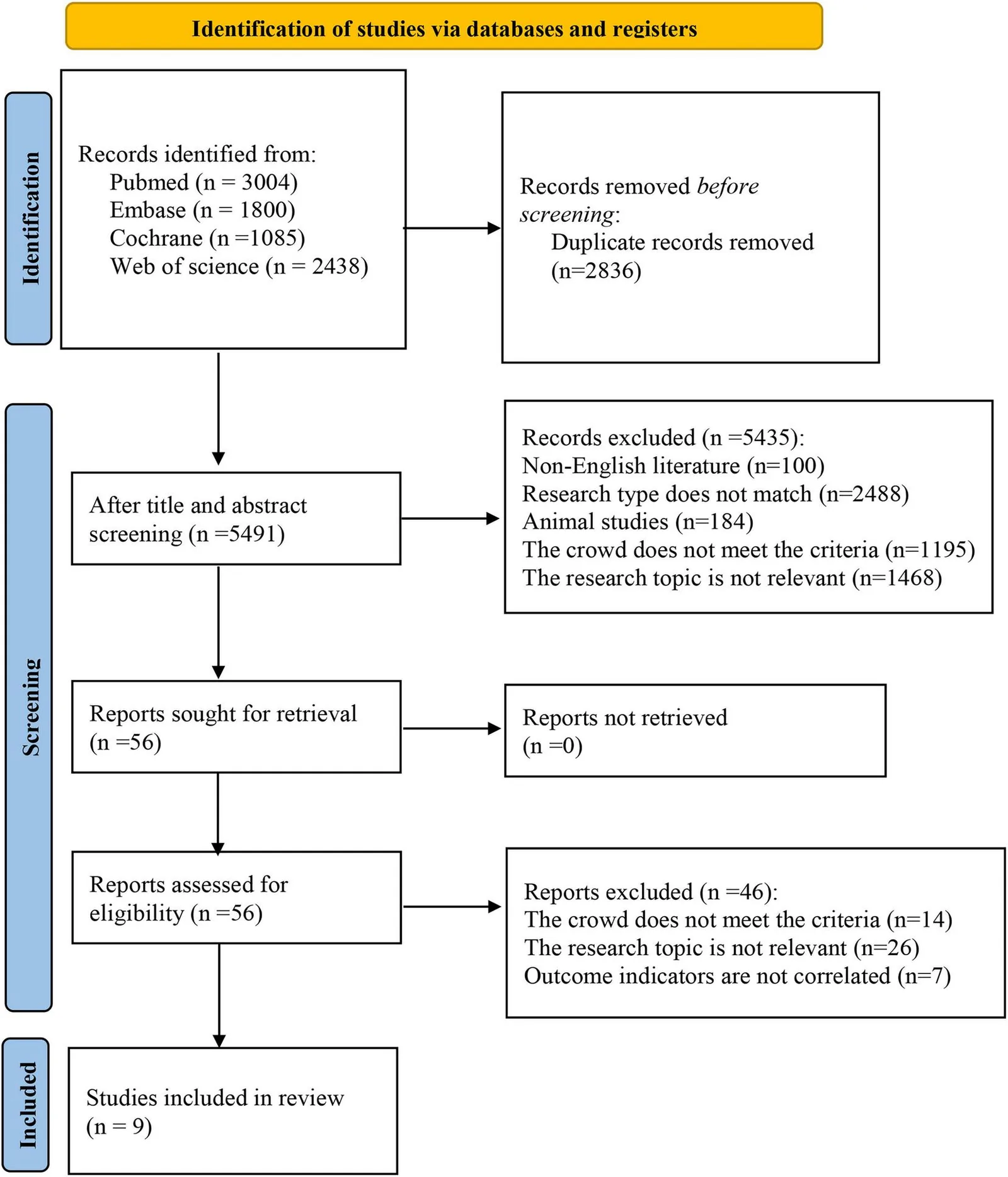
Prisma literature screening flowchart.

A total of 8,327 records were identified through the database search. After removing duplicates, 5,491 records remained for title and abstract screening. Following the exclusion of irrelevant studies, 56 articles were assessed for full-text eligibility. Ultimately, nine meta-analyses (Dhanda et al., 2024; Fu et al., 2022; Lau et al., 2022; Liang et al., 2021; Loughrey et al., 2018; Satheesan et al., 2025; Taljaard et al., 2016; Wei et al., 2017; Yu et al., 2024) were included in this umbrella review (Figure 1).

### 3.2 Characteristics of included meta-analyses

Nine systematic reviews and meta-analyses (Dhanda et al., 2024; Fu et al., 2022; Lau et al., 2022; Liang et al., 2021; Loughrey et al., 2018; Satheesan et al., 2025; Taljaard et al., 2016; Wei et al., 2017; Yu et al., 2024) were included in this umbrella review (Table 1). The number of primary studies per review ranged from 7 to 50, with total sample sizes varying from 681 to over 1.5 million participants. Participant ages spanned from 18 to 65 years in younger adult cohorts to predominantly older adults aged 56–83 years. Hearing loss (HL) assessment varied across reviews, including pure-tone audiometry (PTA), self-reported measures, speech-in-noise tests, whispered voice tests, central auditory tests, and medical records or ICD codes. Central auditory or speech-based hearing assessments were reported in a limited number of included reviews, but these reviews generally combined central and peripheral hearing measures or did not provide separate pooled estimates for central hearing outcomes. The severity of HL was generally classified as mild, moderate, or severe, although one review did not report uniform HL classification. Cognitive outcomes were diverse, including cognitive function, cognitive impairment, dementia, and Alzheimer’s disease. These characteristics demonstrate the heterogeneity of study populations, hearing assessment methods, and cognitive outcomes across the included meta-analyses.

**TABLE 1:** Characteristics of the systematic reviews and meta-analyses included in the umbrella review.

References	Country	Number of studies	Sample size	Age (years)	HL measurement	HL classification	Outcomes
Satheesan et al., 2025	India	7	681	18–65	PTA	Mild-moderate HL	Cognitive function
Dhanda et al., 2024	England	15	21,989	>18	PTA	Mild/moderate/ severe HL	Cognitive impairment; dementia risk
Loughrey et al., 2018	Ireland	40	20,264	>18 (mostly older adults)	PTA	Mild/moderate/ severe HL	Cognitive impairment; dementia risk; Alzheimer
Taljaard et al., 2016	Australia	33	5,735	Mean age ∼57.7	PTA; speech-in-noise tests	Not uniformly reported	Cognitive function
Fu et al., 2022	China	35	372,154	>40 (mostly >60)	PTA; self-reported	Mild/moderate/ severe HL	Cognitive impairment
Yu et al., 2024	England	50	1,548,754	40–83 (mostly older adults)	PTA; self-report: ICD codes; whispered voice test; medical records	Mild/moderate/ severe HL	Cognitive impairment; dementia risk; Alzheimer
Liang et al., 2021	China	14	726,900	56–79	PTA; self-reported: ICD codes; speech recognition tests	Mild/moderate/ severe HL	Dementia risk; Alzheimer
Wei et al., 2017	USA	10	15,521	56.1–77.8	PTA; central auditory tests; whispered voice test	Mild/moderate/ severe HL	Cognitive impairment: Dementia risk
Lau et al., 2022	England	34	48,017	56–76	PTA; self-reported; speech audiometry; central auditory tests	Mild/moderate/ severe HL	Cognitive impairment

PTA, pure-tone audiometry; HL, hearing loss; ICD, International Classification of Diseases.

### 3.3 Quality assessment

**FIGURE 2:**
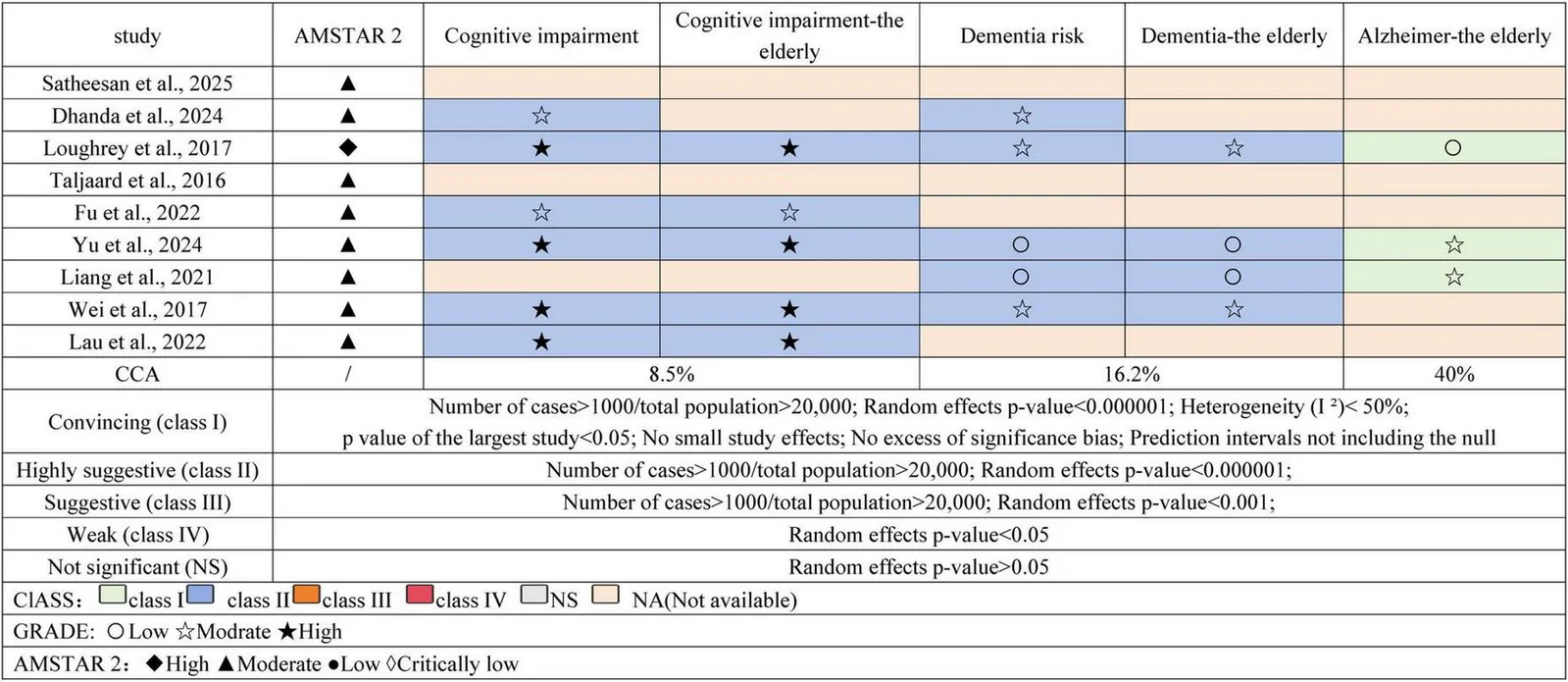
Evidence summary chart.

The methodological quality of the included systematic reviews and meta-analyses was assessed using the 16-item AMSTAR 2 tool. Overall, the majority of studies demonstrated robust methodology across core domains. Most reviews clearly defined research questions and inclusion criteria incorporating PICO components (Item A) and provided a priori description of review methods (Item B). Study design selection, comprehensive literature searches, duplicate study selection and data extraction, and adequate description of included studies were consistently addressed (Items C–H). Risk of bias in individual studies was generally evaluated using standardized tools such as NOS, JBI, or MMAT (Item I). A notable limitation across all reviews was the lack of reporting of funding sources for the included primary studies (Item J, all “No”), reflecting a common gap in observational research. Meta-analytical methods were appropriate (Item K), and risk of bias impact was partially considered in most reviews (Item L, “Partial Yes”), typically via sensitivity analyses rather than formal meta-regression. RoB was generally accounted for in interpreting results (Item M), and heterogeneity was adequately discussed (Item N). Publication bias assessment was inconsistently reported (Item O, mostly “Partial Yes”), while conflicts of interest for the reviews themselves were generally declared (Item P). Based on these findings, the overall methodological quality of the included reviews was moderate to high, with most limitations related to incomplete reporting of primary study funding, partial assessment of RoB impact on meta-analysis results, and incomplete evaluation of publication bias. Detailed item-by-item AMSTAR 2 assessments for each included review, including the rationale for each rating (Yes, No, Partial Yes), are provided in Figure 2 and Supplementary Data Sheet 2.

### 3.4 Overlap of primary studies across meta-analyses

The overlap of primary studies across the included meta-analyses was evaluated using the corrected covered area (CCA). Moderate overlap was observed for cognitive impairment (CCA = 8.5%), whereas extremely high overlap was found for dementia (CCA = 16.2%) and Alzheimer’s disease (CCA = 40%). This pattern reflects the reliance of several meta-analyses on a limited number of shared cohort studies. See Figure 2 and Supplementary Data Sheet 3.

### 3.5 Association between hearing loss and cognitive outcomes

#### 3.5.1 Cognitive impairment

**FIGURE 3:**
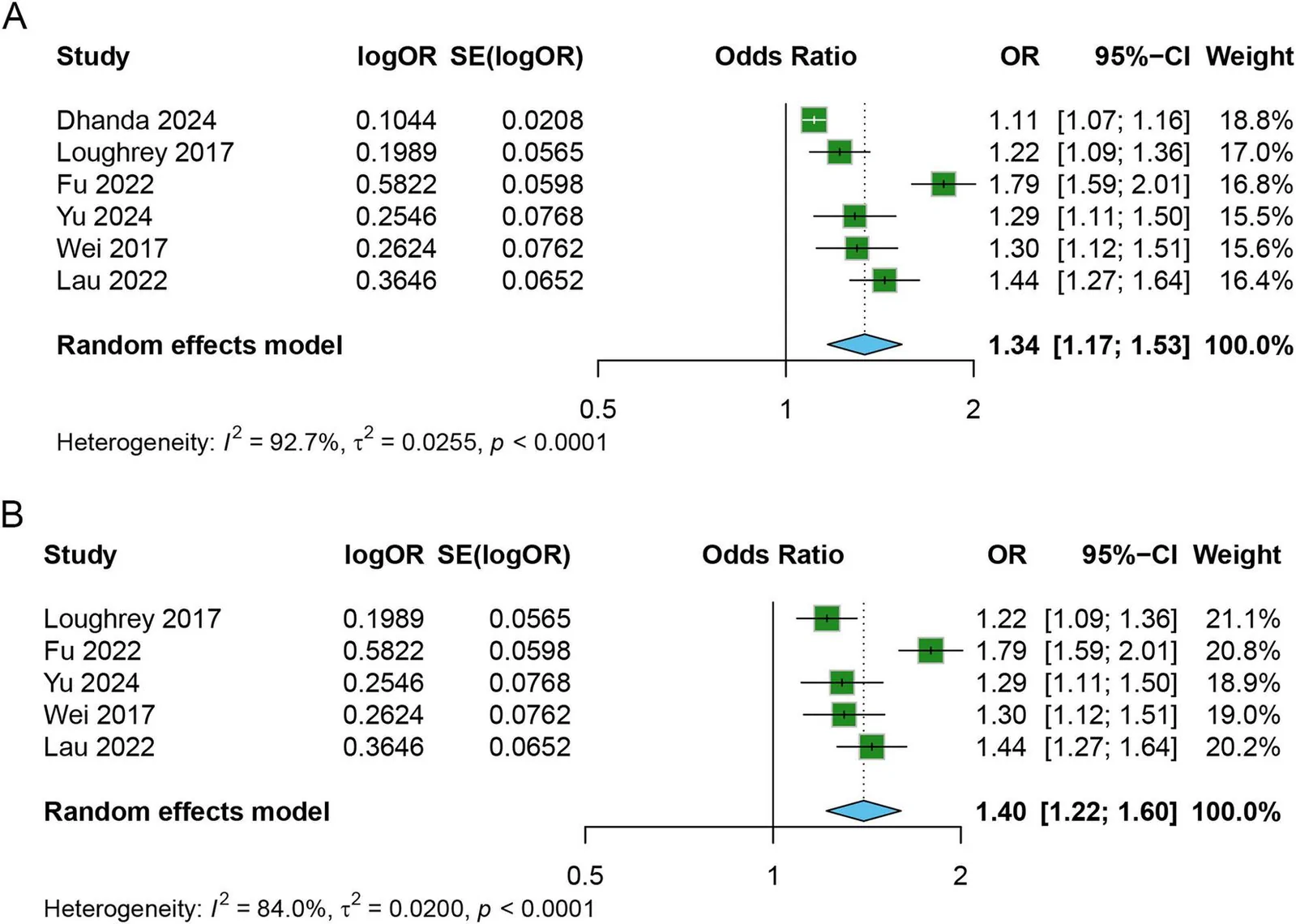
Forest plot of the association between hearing loss and cognitive impairment in overall adults **(A)** and older adults **(B)**.

Six meta-analyses (Dhanda et al., 2024; Fu et al., 2022; Lau et al., 2022; Loughrey et al., 2018; Wei et al., 2017; Yu et al., 2024) evaluated the association between hearing loss and cognitive impairment. In the overall adult population, hearing loss was significantly associated with an increased risk of cognitive impairment, with a pooled effect size of OR = 1.34 (95% CI: 1.17–1.53). Substantial heterogeneity was observed across studies (I^2^ = 92.7%) (Figure 3A). In the subgroup analysis focusing on older adults, five meta-analyses (Fu et al., 2022; Lau et al., 2022; Loughrey et al., 2018; Wei et al., 2017; Yu et al., 2024) were included. The association remained significant, with a pooled OR of 1.40 (95% CI: 1.22–1.60), while heterogeneity was slightly lower (I^2^ = 84.0%) (Figure 3B). These findings suggest that the association between hearing loss and cognitive impairment may be slightly stronger in older populations. Sensitivity analyses indicated that the pooled results were stable, and no single study substantially influenced the overall effect estimate (Supplementary Data Sheets 4A, B).

#### 3.5.2 Dementia

**FIGURE 4:**
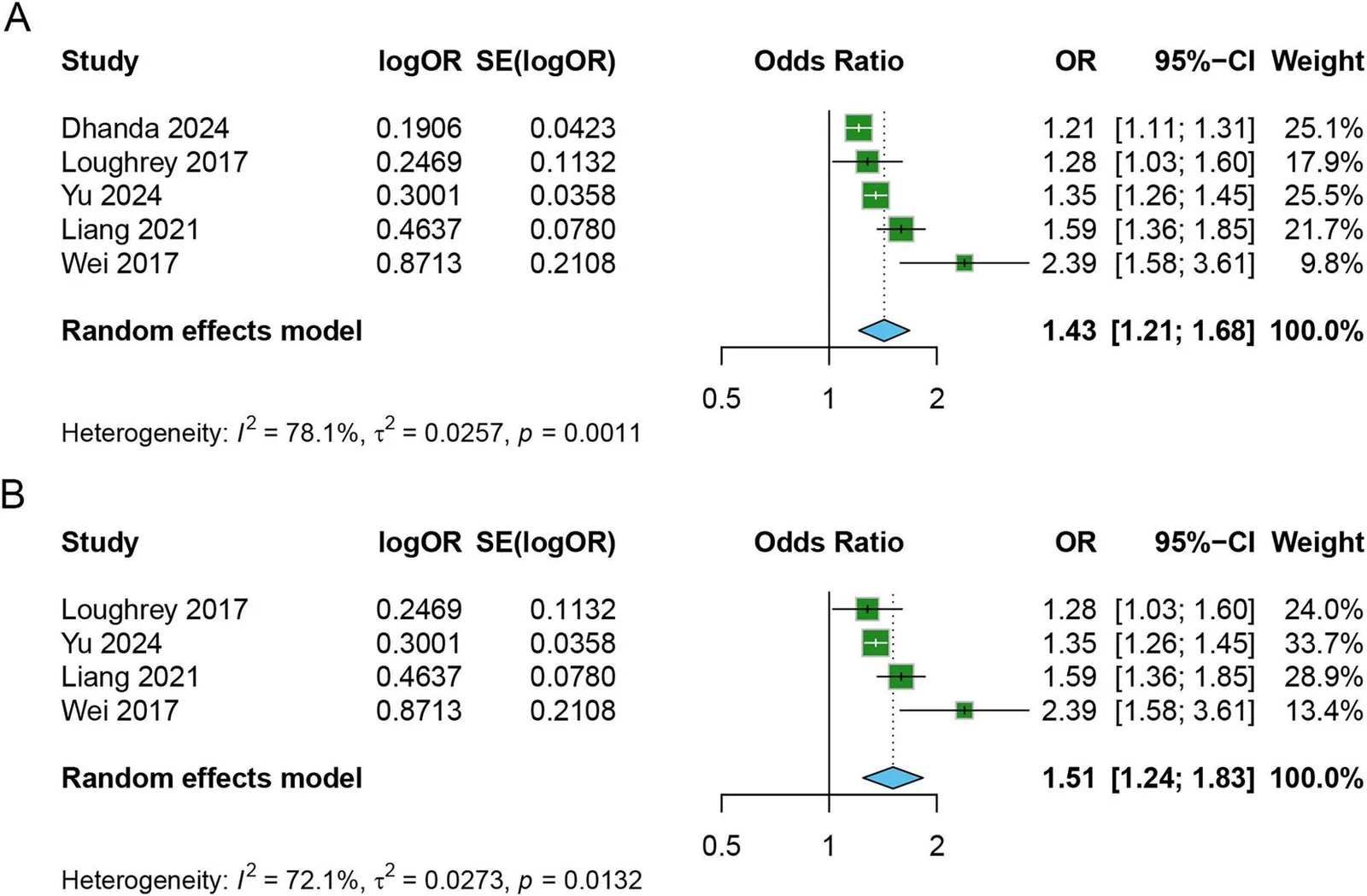
Forest plot of the association between hearing loss and dementia in overall adults **(A)** and older adults **(B)**.

Five meta-analyses (Dhanda et al., 2024; Liang et al., 2021; Loughrey et al., 2018; Wei et al., 2017; Yu et al., 2024) were included to evaluate the association between hearing loss and dementia risk. The pooled analysis using a random-effects model showed that hearing loss was significantly associated with an increased risk of dementia (OR = 1.43, 95% CI: 1.21–1.68), with substantial heterogeneity across studies (I^2^ = 78.1%) (Figure 4A). In the subgroup analysis focusing on older adults, four meta-analyses (Liang et al., 2021; Loughrey et al., 2018; Wei et al., 2017; Yu et al., 2024) primarily involving older populations were included. The pooled effect remained significant (OR = 1.51, 95% CI: 1.24–1.83), with heterogeneity of I^2^ = 72.1% (Figure 4B), suggesting a slightly stronger association in older individuals. Sensitivity analysis in the overall adult population indicated that the results were stable (Supplementary Data Sheet 4C).

#### 3.5.3 Alzheimer’s disease

**FIGURE 5:**
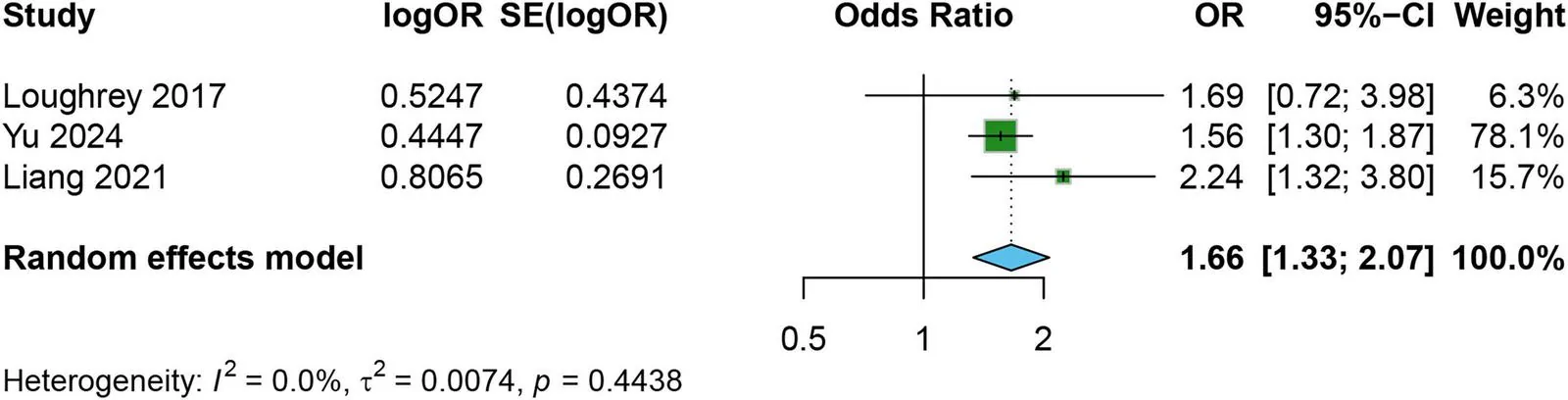
Forest plot of the association between hearing loss and Alzheimer’s disease in older adults.

Three meta-analyses (Liang et al., 2021; Loughrey et al., 2018; Yu et al., 2024) were included to evaluate the association between hearing loss and the risk of Alzheimer’s disease in older adults. The pooled results indicated that hearing loss was significantly associated with an increased risk of Alzheimer’s disease in older individuals (OR = 1.66, 95% CI: 1.33–2.07). Heterogeneity across studies was negligible (I^2^ = 0%) (Figure 5), indicating high consistency among the included meta-analyses. Overall, these findings suggest that hearing loss is an important risk factor for Alzheimer’s disease in older adults, with consistent effect directions across the included studies.

#### 3.5.4 Cognitive function outcomes

Due to differences in cognitive assessment scales and scoring directions across studies, the sign of the standardized mean difference (SMD) was not directly comparable. Therefore, the interpretation focused on the direction of the association rather than the magnitude or sign of the SMD. Two included meta-analyses provided domain-specific cognitive function results. Satheesan et al. (2025) reported that hearing loss was associated with poorer overall cognition (SMD = −0.43, 95% CI: −0.63 to −0.23), memory (SMD = −0.25, 95% CI: −0.45 to −0.06), and executive function (SMD = −0.42, 95% CI: −0.81 to −0.02), where negative values indicated poorer cognitive performance in individuals with hearing loss. Taljaard et al. (2016) further reported poorer performance in general cognition (SMD = 0.21, 95% CI: 0.07 to 0.35), attention/processing speed (SMD = 0.64, 95% CI: 0.41 to 0.87), working memory (SMD = 0.45, 95% CI: 0.29 to 0.61), long-term memory (SMD = 0.40, 95% CI: 0.12 to 0.69), executive function (SMD = 0.24, 95% CI: 0.16 to 0.32), and language ability (SMD = 0.32, 95% CI: 0.19 to 0.44), where positive values indicated poorer cognitive performance in the hearing loss group. Across these domain-specific findings, the most pronounced associations were observed for attention/processing speed, working memory, overall cognition, and executive function, although direct comparison across domains should be made cautiously because the included reviews used different cognitive tests, scoring directions, and study populations. Overall, the available evidence suggests that hearing loss is not limited to a single cognitive outcome but is associated with poorer performance across multiple domains, supporting a multidimensional relationship between hearing impairment and cognitive function (Table 2). Evidence specifically reported as longitudinal cognitive decline was reviewed during data extraction; however, the included meta-analyses reported this construct using heterogeneous definitions, cognitive scales, and effect measures, and therefore a separate quantitative synthesis for longitudinal cognitive decline was not performed.

**TABLE 2:** Meta-analytic results of hearing loss and cognitive function outcomes.

References	Outcome	SMD	95% CI	I^2^ (%)	Included studies	Sample size	Direction
Satheesan et al., 2025	Overall cognition	−0.43	−0.63, −0.23	14	6	636	Worse cognition
Satheesan et al., 2025	Memory	−0.25	−0.45, −0.06	0	4	474	Worse cognition
Satheesan et al., 2025	Executive function	−0.42	−0.81, −0.02	67	4	492	Worse cognition
Taljaard et al., 2016	General cognition	0.21	0.07, 0.35	51.78	5	4260	Worse cognition
Taljaard et al., 2016	Attention/ processing speed	0.64	0.41, 0.87	53.67	10	NR	Worse cognition
Taljaard et al., 2016	Working memory	0.45	0.29, 0.61	61.18	29	NR	Worse cognition
Taljaard et al., 2016	Long-term memory	0.4	0.12, 0.69	72.51	6	NR	Worse cognition
Taljaard et al., 2016	Executive function	0.24	0.16, 0.32	4.29	12	NR	Worse cognition
Taljaard et al., 2016	Language ability	0.32	0.19, 0.44	0	13	NR	Worse cognition

The sign of standardized mean differences (SMD) may differ across studies due to different cognitive test scoring systems: in Satheesan et al. (2025), negative values indicate worse cognition in the hearing loss group, whereas in Taljaard et al. (2016), positive values indicate worse cognition; I^2^ represents the between-study heterogeneity; NR, not reported; the original study did not provide sample size information.

### 3.6 Evidence credibility assessment

The GRADE assessment of the included systematic reviews and meta-analytic outcomes indicated that most outcomes had a low risk of bias and relatively high precision, although moderate to high heterogeneity was observed in some outcomes (I^2^ ranging from 4% to 72%). A few outcomes were upgraded by one level due to large effect sizes (OR/RR ≥ 2 or SMD ≥ 0.5), whereas outcomes with small sample sizes or wide confidence intervals were downgraded. Based on the overall GRADE results, the consistency of effect directions, and statistical significance, the overall evidence from the umbrella meta-analysis was further classified using the Class of Evidence framework. The results showed that the overall evidence for cognitive impairment and dementia was classified as Class II (highly suggestive evidence), indicating a moderate level of credibility, although the relatively high heterogeneity warrants cautious interpretation. For Alzheimer’s disease in older adults, the effect directions were consistent across studies, and the large sample size and narrow confidence intervals resulted in evidence approaching Class I (convincing evidence), although further high-quality evidence is still needed to confirm this level of certainty. For cognitive function outcomes, the certainty of evidence varied across different cognitive domains, ranging from low to high. Overall, the available evidence consistently supports an association between hearing loss and both cognitive impairment and dementia; however, interpretation of specific outcomes should consider the presence of heterogeneity and limitations in sample size. Detailed results are presented in Figure 2 and Supplementary Data Sheet 5.

## 4 Discussion

This umbrella meta-analysis systematically synthesized evidence from existing systematic reviews and meta-analyses to evaluate the association between hearing loss and cognitive outcomes in adults. Overall, hearing loss was consistently associated with increased risks of cognitive impairment, dementia, and Alzheimer’s disease. Although the subgroup analyses suggested slightly stronger associations among older adults, these findings should be interpreted as supportive rather than as the primary novel contribution of this review. The main contribution of the present study lies in providing an updated synthesis of recent meta-analytic evidence, evaluating the independence and credibility of the evidence through overlap and certainty assessments, and summarizing cognitive-domain findings where available. Across different cognitive domains and assessment tools, the direction of associations remained largely consistent, suggesting that hearing loss is broadly linked to multidimensional cognitive decline.

The present findings reinforce prior evidence supporting a robust association between hearing loss and cognitive decline (Liang et al., 2021; Lin et al., 2013; Loughrey et al., 2018). Notably, stronger effect estimates observed in older adults suggest that age may act as an important effect modifier. From the perspective of aging-related theories, reduced cognitive reserve and diminished neural plasticity may limit compensatory capacity in response to sensory deficits (Stern, 2012). In parallel, age-related neurodegenerative changes, including synaptic loss and cortical atrophy, may increase vulnerability to the adverse effects of auditory deprivation (Kolo et al., 2025).

Several mechanisms have been proposed to explain this relationship. The sensory deprivation hypothesis suggests that reduced auditory input may accelerate cortical atrophy (Stern, 2012), while the cognitive load hypothesis posits that increased listening effort may divert cognitive resources away from higher-order processing (Kolo et al., 2025). In addition, reduced social engagement associated with hearing impairment may further contribute to cognitive decline (Dawes et al., 2015; Zhao et al., 2024). Importantly, hearing loss and cognitive decline may also share common pathological mechanisms, including vascular dysfunction, chronic inflammation, and neurodegeneration (Bisogno et al., 2021), indicating that their relationship likely reflects a complex interplay of biological and behavioral factors.

The stronger associations observed in older populations may be explained by several converging factors. With advancing age, compensatory mechanisms become progressively impaired, reducing the ability to offset sensory deficits. At the same time, the accumulation of multimorbidity, particularly cardiometabolic conditions such as cardiovascular disease and diabetes, may exacerbate both hearing loss and cognitive decline through shared vascular and metabolic pathways (Wang et al., 2022). In addition, age-related changes in social networks, including social isolation and reduced participation, may further amplify the cognitive consequences of hearing impairment (Araki, 2025).

Notably, a potential bidirectional relationship between hearing loss and early neurodegenerative processes should also be considered. Emerging evidence suggests that early stages of neurodegenerative diseases, such as Alzheimer’s disease, may, at least in part, affect central auditory processing, leading to apparent hearing difficulties (Kolo et al., 2025). In the present umbrella review, central auditory or speech-based hearing assessments were identified in some included reviews; however, the available meta-analytic evidence did not consistently report central hearing loss as a separate exposure category with independent effect estimates for cognitive decline, cognitive impairment, dementia, or Alzheimer’s disease. Therefore, a separate quantitative synthesis for central hearing loss was not feasible. Conversely, untreated peripheral or mixed hearing loss may accelerate neurodegenerative changes through reduced sensory input and increased cognitive load. This bidirectional interaction has important clinical implications, as hearing impairment may serve not only as a modifiable risk factor but also as an early clinical marker of cognitive decline.

Substantial heterogeneity was observed in the outcomes of cognitive impairment and dementia. This heterogeneity may be attributable to differences in hearing loss assessment methods (e.g., pure-tone audiometry, self-reported measures, or medical records), variability in the definitions of cognitive outcomes, and differences in participant characteristics across studies. Furthermore, variations in study design, follow-up duration, and adjustment for confounding factors may have influenced the pooled estimates (Bisogno et al., 2021). These methodological and clinical differences should be carefully considered when interpreting the overall findings.

The domain-specific findings provide additional insight into the pattern of cognitive involvement associated with hearing loss. Although quantitative re-meta-analysis across cognitive domains was not appropriate because of differences in cognitive assessment tools, scoring directions, and study populations, the qualitative synthesis showed a broadly consistent association between hearing loss and poorer cognitive performance. The available evidence suggested that attention/processing speed, working memory, executive function, memory, language ability, and overall cognition may all be affected, with relatively larger standardized differences reported for attention/processing speed and working memory in one included meta-analysis. These findings suggest that hearing loss may be related to multidimensional cognitive vulnerability rather than to impairment in a single cognitive domain. However, because the number of domain-specific meta-analyses was limited and the cognitive tests were heterogeneous, these results should be interpreted as exploratory and hypothesis-generating. Similarly, longitudinal cognitive decline was considered within the broader cognitive-outcome framework, but the available meta-analytic evidence did not provide sufficiently consistent definitions or effect estimates to support a separate pooled analysis.

From an evidence synthesis perspective, this study extends previous meta-analyses by integrating multiple methodological evaluation frameworks, including AMSTAR-2, GRADE, and evidence credibility classification. Overall, the methodological quality of the included reviews was moderate to high. However, several limitations were identified, including incomplete reporting of publication bias, limited assessment of the impact of bias on pooled estimates, and insufficient reporting of funding sources in primary studies. In addition, CCA analysis revealed a moderate degree of overlap in primary studies across the included meta-analyses, particularly for dementia and Alzheimer’s disease outcomes. Therefore, the potential influence of overlapping evidence should be taken into account when interpreting the strength and independence of the findings.

From a clinical and public health perspective, these findings have important implications. Given the high prevalence of hearing loss among middle-aged and older adults, hearing health should be formally incorporated into comprehensive geriatric assessment frameworks and primary prevention strategies for cognitive impairment (Powell et al., 2022), particularly within aging populations. Early identification and management of hearing impairment may facilitate risk stratification and timely intervention for individuals at high risk of cognitive decline. Importantly, intervention during midlife—prior to the onset of significant cognitive deterioration—may offer greater neuroprotective benefits compared to interventions initiated in later life (Machado-Fragua et al., 2025).

Emerging evidence also suggests that hearing interventions, particularly the use of hearing aids, may be associated with a reduced risk of cognitive decline (Yeo et al., 2023), although the magnitude and causality of this effect remain to be fully established. Future research should prioritize well-designed prospective studies and randomized controlled trials to determine whether treating hearing loss can prevent or delay cognitive impairment and dementia, and to identify the optimal timing and target populations for such interventions.

### 4.1 Limitations

Several limitations should be acknowledged. First, as this umbrella review was based on previously published systematic reviews and meta-analyses, the findings remain dependent on the quality and methodological variability of the original studies. Second, heterogeneity in exposure definitions and outcome measurements may have contributed to variability in the results. Third, overlap in primary studies across meta-analyses may affect the independence of the evidence. In addition, most included evidence was derived from observational studies, limiting causal inference due to potential reverse causality and residual confounding. In addition, the restriction to English-language publications may have introduced potential language bias. Finally, although central auditory processing and central hearing measures were reported in some included reviews, the lack of separately reported pooled estimates limited our ability to evaluate central hearing loss as an independent exposure.

### 4.2 Strengths

Despite these limitations, this study has several notable strengths. As an umbrella meta-analysis, it provides a higher-level synthesis of evidence by integrating findings from multiple meta-analyses. The incorporation of methodological quality assessment, overlap analysis, and evidence credibility classification enhances the robustness and interpretability of the findings. In the context of rapidly growing but heterogeneous evidence, this study offers a comprehensive and systematic evaluation of the association between hearing loss and cognitive outcomes.

## 5 Conclusion

This umbrella meta-analysis indicates that hearing loss in adults is significantly associated with increased risks of cognitive impairment, dementia, and Alzheimer’s disease, with stronger associations observed in older populations. The available evidence generally supports hearing loss as an important correlate of cognitive decline. However, due to heterogeneity across studies, overlap of primary studies, and the inherent limitations of observational evidence, causal interpretations should be made with caution. Future research should focus on well-designed prospective studies with more standardized definitions, better control of confounding factors, and longer follow-up periods. In addition, further studies are needed to evaluate the potential protective effects of hearing interventions on cognitive outcomes, thereby providing stronger evidence to inform dementia prevention strategies and promote healthy aging at the population level.
