# Supplementary material for: The association between hearing loss in adults and cognitive outcomes: an umbrella review and meta-analysis
Source: Front Aging Neurosci. 2026 Aug 24;18:1852163. doi: 10.3389/fnagi.2026.1852163 (PMC13548122; doi:10.3389/fnagi.2026.1852163)
Supplement: Supplementary file 1 [file Data_Sheet_1.docx]

**Menu**

**[Supplementary 1: Search Strategy	2](#_Toc22639)**

**[Supplementary 2: AMSTAR2 Evaluation Details	7](#_Toc15288)**

**[Supplementary 3:Overlap of primary studies across included meta-analyses (CCA analysis)	17](#_Toc19153)**

**[Supplementary 4: Sensitivity analysis	2](#_Toc2619)0**

**[Supplementary 5: GRADE assessment and CLASS of evidence for the association between hearing loss and cognitive outcomes](#_Toc6608)	21**

# Supplementary 1: Search Strategy

# **Pubmed**

| Search | Query | Search Details | Results |
| --- | --- | --- | --- |
| 1 | ((((((((((((((((((((((Hearing Loss[MeSH Terms]) OR (Hearing Loss[Title/Abstract])) OR (Hearing Impairment[Title/Abstract])) OR (Hypoacus*[Title/Abstract])) OR (Transitory Deafness*[Title/Abstract])) OR (Transitory Hearing Loss*[Title/Abstract])) OR (auditory defect[Title/Abstract])) OR (deaf[Title/Abstract])) OR (deafness[Title/Abstract])) OR (hard of hearing[Title/Abstract])) OR (hearing damage[Title/Abstract])) OR (hearing defect[Title/Abstract])) OR (hearing difficulty[Title/Abstract])) OR (hypacousia[Title/Abstract])) OR (hypacousis[Title/Abstract])) OR (hypacusia[Title/Abstract])) OR (hypacusis[Title/Abstract])) OR (hypakousia[Title/Abstract])) OR (hypakusis[Title/Abstract])) OR (hypoacousia[Title/Abstract])) OR (hypoacousis[Title/Abstract])) OR (hypoakusis[Title/Abstract])) OR (impaired hearing[Title/Abstract]) | "hearing loss"[MeSH Terms] OR "hearing loss"[Title/Abstract] OR "hearing impairment"[Title/Abstract] OR "hypoacus*"[Title/Abstract] OR "transitory deafness*"[Title/Abstract] OR "transitory hearing loss*"[Title/Abstract] OR "auditory defect"[Title/Abstract] OR "deaf"[Title/Abstract] OR "deafness"[Title/Abstract] OR "hard of hearing"[Title/Abstract] OR "hearing damage"[Title/Abstract] OR "hearing defect"[Title/Abstract] OR "hearing difficulty"[Title/Abstract] OR "hypacousia"[Title/Abstract] OR "hypacousis"[Title/Abstract] OR "hypacusia"[Title/Abstract] OR "hypacusis"[Title/Abstract] OR "hypakousia"[Title/Abstract] OR "hypoacousia"[Title/Abstract] OR "hypoacousis"[Title/Abstract] OR "impaired hearing"[Title/Abstract] | 127,958 |
| 2 | (((((((((((("Cognition"[Mesh]) OR (Cognition[Title/Abstract])) OR (Cognitive Function[Title/Abstract])) OR (Function, Cognitive[Title/Abstract])) OR (cognitive accessibility[Title/Abstract])) OR (cognitive balance[Title/Abstract])) OR (cognitive dissonance[Title/Abstract])) OR (cognitive structure[Title/Abstract])) OR (cognitive symptoms[Title/Abstract])) OR (cognitive task[Title/Abstract])) OR (cognitive thinking[Title/Abstract])) OR (neurobehavioural manifestations[Title/Abstract])) OR (volition[Title/Abstract]) | "Cognition"[MeSH Terms] OR "Cognition"[Title/Abstract] OR "cognitive function"[Title/Abstract] OR "function cognitive"[Title/Abstract] OR "cognitive accessibility"[Title/Abstract] OR "cognitive balance"[Title/Abstract] OR "cognitive dissonance"[Title/Abstract] OR "cognitive structure"[Title/Abstract] OR "cognitive symptoms"[Title/Abstract] OR "cognitive task"[Title/Abstract] OR "cognitive thinking"[Title/Abstract] OR "neurobehavioural manifestations"[Title/Abstract] OR "volition"[Title/Abstract] | 362,345 |
| 3 | (((((((((((((((((((((((Hearing Loss[MeSH Terms]) OR (Hearing Loss[Title/Abstract])) OR (Hearing Impairment[Title/Abstract])) OR (Hypoacus*[Title/Abstract])) OR (Transitory Deafness*[Title/Abstract])) OR (Transitory Hearing Loss*[Title/Abstract])) OR (auditory defect[Title/Abstract])) OR (deaf[Title/Abstract])) OR (deafness[Title/Abstract])) OR (hard of hearing[Title/Abstract])) OR (hearing damage[Title/Abstract])) OR (hearing defect[Title/Abstract])) OR (hearing difficulty[Title/Abstract])) OR (hypacousia[Title/Abstract])) OR (hypacousis[Title/Abstract])) OR (hypacusia[Title/Abstract])) OR (hypacusis[Title/Abstract])) OR (hypakousia[Title/Abstract])) OR (hypakusis[Title/Abstract])) OR (hypoacousia[Title/Abstract])) OR (hypoacousis[Title/Abstract])) OR (hypoakusis[Title/Abstract])) OR (impaired hearing[Title/Abstract])) AND ((((((((((((("Cognition"[Mesh]) OR (Cognition[Title/Abstract])) OR (Cognitive Function[Title/Abstract])) OR (Function, Cognitive[Title/Abstract])) OR (cognitive accessibility[Title/Abstract])) OR (cognitive balance[Title/Abstract])) OR (cognitive dissonance[Title/Abstract])) OR (cognitive structure[Title/Abstract])) OR (cognitive symptoms[Title/Abstract])) OR (cognitive task[Title/Abstract])) OR (cognitive thinking[Title/Abstract])) OR (neurobehavioural manifestations[Title/Abstract])) OR (volition[Title/Abstract])) | ("hearing loss"[MeSH Terms] OR "hearing loss"[Title/Abstract] OR "hearing impairment"[Title/Abstract] OR "hypoacus*"[Title/Abstract] OR "transitory deafness*"[Title/Abstract] OR "transitory hearing loss*"[Title/Abstract] OR "auditory defect"[Title/Abstract] OR "deaf"[Title/Abstract] OR "deafness"[Title/Abstract] OR "hard of hearing"[Title/Abstract] OR "hearing damage"[Title/Abstract] OR "hearing defect"[Title/Abstract] OR "hearing difficulty"[Title/Abstract] OR "hypacousia"[Title/Abstract] OR "hypacousis"[Title/Abstract] OR "hypacusia"[Title/Abstract] OR "hypacusis"[Title/Abstract] OR "hypakousia"[Title/Abstract] OR "hypoacousia"[Title/Abstract] OR "hypoacousis"[Title/Abstract] OR "impaired hearing"[Title/Abstract]) AND ("Cognition"[MeSH Terms] OR "Cognition"[Title/Abstract] OR "cognitive function"[Title/Abstract] OR "function cognitive"[Title/Abstract] OR "cognitive accessibility"[Title/Abstract] OR "cognitive balance"[Title/Abstract] OR "cognitive dissonance"[Title/Abstract] OR "cognitive structure"[Title/Abstract] OR "cognitive symptoms"[Title/Abstract] OR "cognitive task"[Title/Abstract] OR "cognitive thinking"[Title/Abstract] OR "neurobehavioural manifestations"[Title/Abstract] OR "volition"[Title/Abstract]) | 3,004 |

**Embase**

| No. | Query | Results |
| --- | --- | --- |
| #1 | 'hearing loss':ab,ti OR 'hearing impairment':ab,ti OR hypoacus*:ab,ti OR 'transitory deafness*':ab,ti OR 'transitory hearing loss*':ab,ti OR 'auditory defect':ab,ti OR deaf:ab,ti OR deafness:ab,ti OR 'hard of hearing':ab,ti OR 'hearing damage':ab,ti OR 'hearing defect':ab,ti OR 'hearing difficulty':ab,ti OR hypacousia:ab,ti OR hypacousis:ab,ti OR hypacusia:ab,ti OR hypacusis:ab,ti OR hypakousia:ab,ti OR hypakusis:ab,ti OR hypoacousia:ab,ti OR hypoacousis:ab,ti OR hypoakusis:ab,ti OR 'impaired hearing':ab,ti | 133773 |
| #2 | cognition:ab,ti OR 'cognitive function':ab,ti OR 'function, cognitive':ab,ti OR 'cognitive accessibility':ab,ti OR 'cognitive balance':ab,ti OR 'cognitive dissonance':ab,ti OR 'cognitive structure':ab,ti OR 'cognitive symptoms':ab,ti OR 'cognitive task':ab,ti OR 'cognitive thinking':ab,ti OR 'neurobehavioural manifestations':ab,ti OR volition:ab,ti | 256938 |
| #3 | #1 AND #2 | 1800 |

**Cochrane**

| ID | Search | Hits |
| --- | --- | --- |
| #1 | MeSH descriptor: [Hearing Loss] explode all trees | 2005 |
| #2 | (Hearing Loss):ti,ab,kw OR (Hearing Impairment):ti,ab,kw OR (Hypoacus*):ti,ab,kw OR (Transitory Deafness*):ti,ab,kw OR (Transitory Hearing Loss*):ti,ab,kw | 5768 |
| #3 | (auditory defect):ti,ab,kw OR (deaf):ti,ab,kw OR (deafness):ti,ab,kw OR (hard of hearing):ti,ab,kw OR (hearing damage):ti,ab,kw | 2424 |
| #4 | (hearing defect):ti,ab,kw OR (hearing difficulty):ti,ab,kw OR (hypacousia):ti,ab,kw OR (hypacousis):ti,ab,kw OR (hypacusia):ti,ab,kw | 427 |
| #5 | (hypacusis):ti,ab,kw OR (hypakousia):ti,ab,kw OR (hypakusis):ti,ab,kw OR (hypoacousia):ti,ab,kw OR (hypoacousis):ti,ab,kw | 8 |
| #6 | (hypoakusis):ti,ab,kw OR (impaired hearing):ti,ab,kw | 656 |
| #7 | #1 or #2 or #3 or #4 or #5 or #6 | 7333 |
| #8 | MeSH descriptor: [Cognition] explode all trees | 17076 |
| #9 | (Cognition):ti,ab,kw OR (Cognitive Function):ti,ab,kw OR (Function, Cognitive):ti,ab,kw OR (cognitive accessibility):ti,ab,kw OR (cognitive balance):ti,ab,kw | 62714 |
| #10 | (cognitive dissonance):ti,ab,kw OR (cognitive structure):ti,ab,kw OR (cognitive symptoms):ti,ab,kw OR (cognitive task):ti,ab,kw OR (cognitive thinking):ti,ab,kw | 41003 |
| #11 | (neurobehavioural manifestations):ti,ab,kw OR (volition):ti,ab,kw | 317 |
| #12 | #8 or #9 or #10 or #11 | 87648 |
| #13 | #7 and #12 | 1085 |

**Wos**

| # | Search Query | Results |
| --- | --- | --- |
| 1 | TS=("Hearing Loss") OR TS=("Hearing Impairment") OR TS=("Hypoacus*") OR TS=("Transitory Deafness*") OR TS=("Transitory Hearing Loss*") OR TS=("auditory defect") OR TS=(deaf*) OR TS=("hard of hearing") OR TS=("hearing damage") OR TS=("hearing defect") OR TS=("hearing difficulty") OR TS=(hypacous*) OR TS=(hypacus*) OR TS=(hypoacous*) OR TS=(hypoacus*) OR TS=(hypakous*) OR TS=(hypakus*) OR TS=(hypoakus*) OR TS=("impaired hearing") | 125162 |
| 2 | TS=("Cognition") OR TS=("Cognitive Function") OR TS=("Function, Cognitive") OR TS=("cognitive accessibility") OR TS=("cognitive balance") OR TS=("cognitive dissonance") OR TS=("cognitive structure") OR TS=("cognitive symptoms") OR TS=("cognitive task") OR TS=("cognitive thinking") OR TS=("neurobehavioural manifestations") OR TS=("volition") | 337345 |
| 3 | #1 AND #2 | 2438 |

# Supplementary 2: AMSTAR2 Evaluation Details

| **Item** | **AMSTAR 2 question** |
| --- | --- |
| A | Did the research questions and inclusion criteria include the components of PICO? |
| B | Did the report state that review methods were established prior to conducting the review and justify deviations from the protocol? |
| B | Did the review authors explain their selection of study designs for inclusion? |
| D | Did the review authors use a comprehensive literature search strategy? |
| E | Did the review authors perform study selection in duplicate? |
| F | Did the review authors perform data extraction in duplicate? |
| G | Did the review authors provide a list of excluded studies with justification? |
| H | Did the review authors describe included studies in adequate detail? |
| I | Did the review authors use a satisfactory technique for assessing the risk of bias in individual studies? |
| J | Did the review authors report sources of funding for the included studies? |
| K | If meta-analysis was performed, did the review authors use appropriate statistical methods? |
| L | If meta-analysis was performed, did the review authors assess the potential impact of risk of bias on the results? |
| M | Did the review authors account for risk of bias when interpreting the results? |
| N | Did the review authors explain and discuss any heterogeneity in results? |
| O | Did the review authors investigate publication bias and discuss its likely impact? |
| P | Did the review authors report any potential conflicts of interest, including funding for the review? |

| 1. **Satheesan et al., 2025** | | |
| --- | --- | --- |
| **Item** | **Rating (Y / N / PY)** | **Comments** |
| A | Y | Population (18–65 years), Exposure (hearing loss), Comparison (normal hearing), Outcome (cognitive domains) clearly specified |
| B | Y | Registered in PROSPERO (CRD42025628016); no deviations reported |
| B | Y | Included RCTs, cohort, case-control, and cross-sectional studies; rationale explained in Methods |
| D | Y | Searched PubMed, Scopus, Web of Science, EMBASE, plus backward/forward citation tracking; full search strings in Appendix A |
| E | Y | Two reviewers independently screened titles, abstracts, and full texts; disagreements resolved by a third reviewer |
| F | Y | Two reviewers independently extracted data using piloted Excel form; discrepancies resolved by discussion |
| G | Y | PRISMA flow diagram lists 161 full-text exclusions with reasons (Figure 1) |
| H | Y | Table 1 and text include sample size, age, hearing loss severity, cognitive domains, and cognitive tests |
| I | Y | Used JBI Critical Appraisal Checklist; Table 2 shows individual study ratings |
| J | N | Funding sources for primary studies not reported; only authors’ own funding declared as none |
| K | Y | Random-effects model, Cohen’s d computed correctly, I² and 95% CI reported |
| L | PY | Sensitivity analysis performed, but no formal meta-regression or subgroup analysis by RoB |
| M | Y | Discussion addresses study quality and potential bias impact on results |
| N | Y | I² reported, heterogeneity discussed (0–74%); potential sources explained |
| O | N | No funnel plot or Egger/Begg test; publication bias not formally assessed |
| P | Y | Declared no conflicts of interest; review received no external funding |
| **Quality Rating:Moderate** | | |

Note:Y=yes N=No PY=YS

| 2. **Dhanda et al., 2024** | | |
| --- | --- | --- |
| **Item** | **Rating (Y / N / PY)** | **Comments** |
| A | Y | Clearly defined Population (adults ≥18), Exposure (hearing loss), Outcome (cognitive impairment/dementia), Comparison (normal hearing) |
| B | Y | Registered in PROSPERO; search strategy and protocol documented, no deviations reported |
| B | Y | Included longitudinal cohort studies; rationale provided; cross-sectional and self-reported hearing studies excluded |
| D | Y | Searched Web of Science, PubMed, Scopus, EMBASE, PsychInfo, ProQuest (Dissertations), plus backward/forward citations; no language/date restrictions |
| E | Y | Two reviewers independently screened titles, abstracts, and full texts; disagreements resolved by discussion |
| F | PY | Primary extraction by one reviewer, a second reviewer checked a subset of extracted data; not full independent duplicate extraction |
| G | Y | PRISMA flow diagram and exclusion reasons clearly reported (Figure 2) |
| H | Y | Tables 1–3 provide study characteristics, participants, outcomes, confounders, analysis methods; narrative synthesis included |
| I | Y | Used Item Bank for Assessment of Risk of Bias and Precision for Observational Studies; red-amber-green RAG rating reported for each study |
| J | N | Funding sources for included primary studies not reported |
| K | Y | Meta-analysis of 5 studies for cognitive impairment/dementia; fixed-effects inverse variance method; HRs pooled correctly; heterogeneity assessed with I² |
| L | PY | Only studies not rated high risk of bias were included; narrative discussion of bias provided, but no formal subgroup or meta-regression by RoB |
| M | Y | Discussion acknowledges limitations due to study quality, heterogeneity, measurement differences, and attrition |
| N | Y | I² reported (e.g., 61% for dementia), differences in follow-up, hearing definitions, and outcomes discussed as sources of heterogeneity |
| O | N | No funnel plot or formal tests for small-study bias conducted |
| P | Y | Authors declared no conflicts of interest; review received no external funding |
| **Quality Rating:Moderate** | | |

| 3. **Loughrey et al., 2017** | | |
| --- | --- | --- |
| **Item** | **Rating (Y / N / PY)** | **Comments** |
| A | Y | Population: adults ≥18; Exposure: age-related hearing loss (ARHL); Comparison: normal hearing; Outcomes: cognitive function, cognitive impairment, dementia |
| B | PY | Protocol mentioned but no formal PROSPERO registration; methodology described a priori, deviations not fully detailed |
| B | Y | Included cross-sectional and cohort studies; case-control studies excluded due to higher risk of bias, rationale explained in Methods |
| D | Y | Searched PubMed, EMBASE, SCOPUS, Cochrane Library; also cross-referencing and personal files; keywords and strategy detailed in supplement |
| E | Y | Two reviewers independently screened titles, abstracts, and full texts; discrepancies resolved by discussion or third reviewer |
| F | Y | Two reviewers independently extracted data; consensus or arbitration used to resolve conflicts |
| G | Y | PRISMA flowchart (Figure 1) lists reasons for exclusion for all full-text screened studies |
| H | Y | Tables 1–2 provide study characteristics, participants, cognitive outcomes, audiometric measures, and covariates; narrative description included |
| I | Y | Used STROBE checklist to assess quality; scores provided for all included studies |
| J | N | Funding sources for individual studies not reported |
| K | Y | Random-effects meta-analysis used; continuous outcomes converted to correlation coefficients, categorical outcomes to ORs; I² reported |
| L | Y | Studies with low/acceptable STROBE scores prioritized; sensitivity analyses performed; RoB considered in interpretation |
| M | Y | Discussion section acknowledges study quality and limitations, interprets results cautiously |
| N | Y | Heterogeneity quantified using I² and Q tests; sources of heterogeneity discussed (demographics, audiometric factors, cognitive measures) |
| O | Y | Funnel plots and Egger test performed for outcomes with ≥10 studies; discussed impact on results |
| P | Y | Conflicts of interest declared; review funding sources reported (DeafHear, Irish Research Council, NIH, Atlantic Philanthropies) |
| **Quality Rating:High** | | |

| 4. **Taljaard et al., 2016** | | |
| --- | --- | --- |
| **Item** | **Rating (Y / N / PY)** | **Comments** |
| A | Y | Population: adults; Exposure: untreated/treated hearing impairment; Comparison: normal hearing; Outcomes: general cognition and cognitive domains clearly defined |
| B | PY | Protocol registered with PROSPERO (CRD42013006163), initially for intervention studies; expanded later to include comparative and association studies; deviations explained but not in full detail |
| B | Y | Included cohort, cross-sectional, and pre/post intervention studies; rationale for excluding some study types provided |
| D | Y | Searched PubMed, CINAHL, EMBASE, PsycINFO, Scopus, Academic Search Premier, Cochrane Library, and grey literature (OpenGrey, WHO ICTR); references and author contacts also searched |
| E | Y | Three reviewers independently screened titles and abstracts; discrepancies discussed and resolved; full-text assessment independently by multiple reviewers |
| F | Y | Data extracted and coded for all studies; cross-checked for accuracy; discrepancies resolved by consensus |
| G | Y | PRISMA flowchart with number of excluded full-texts and reasons clearly reported |
| H | Y | Tables 1–2 include sample size, age, cognitive domain, hearing test, intervention status; narrative description provided |
| I | PY | Risk of bias assessment mentioned, mainly focused on study design and sample characteristics; no standardized RoB tool applied across all studies |
| J | N | Funding sources for individual primary studies not reported; review funding declared (Cochlear Foundation, Cochlear Australia) |
| K | Y | Random-effects model used; Cohen’s d and Hedges’s g calculated; heterogeneity (I²) reported; moderator analyses performed |
| L | PY | RoB discussed qualitatively in narrative; no formal subgroup/meta-regression by bias level performed |
| M | Y | Limitations and potential biases discussed in the Discussion section; results interpreted cautiously |
| N | Y | Heterogeneity reported using I² and Q statistics; sources (task type, treatment duration, age) explored and discussed |
| O | Y | Funnel plots and Duval & Tweedie’s trim-and-fill used for outcomes with evidence of bias; discussion included |
| P | Y | Conflicts of interest declared; review supported by Cochlear Foundation and Cochlear Australia |
| **Quality Rating:Moderate** | | |

| 5. **Fu et al., 2022** | | |
| --- | --- | --- |
| **Item** | **Rating (Y / N / PY)** | **Comments** |
| A | Y | Population: adults ≥40 years; Exposure: age-related hearing loss; Comparison: normal hearing; Outcomes: cognitive function, impairment, or dementia clearly defined |
| B | Y | PROSPERO registered (CRD42021235310); protocol predefined, no major deviations reported |
| B | Y | Included RCTs, cohort, cross-sectional, case-control studies; excluded studies without control groups or prior cognitive disorders, rationale provided |
| D | Y | Searched Embase, MEDLINE, Web of Science, PsycINFO, SinoMed, CBM, Google Scholar; English and Chinese publications included; MeSH and keywords described |
| E | Y | Two independent reviewers screened titles, abstracts, and full texts; discrepancies resolved by discussion with third author |
| F | Y | Two reviewers independently extracted data; discrepancies resolved through discussion |
| G | Y | PRISMA flow diagram included (Figure 1) showing excluded studies and reasons |
| H | Y | Table 1 summarizes study characteristics, hearing/cognitive assessments, effect sizes, and participant demographics; narrative description included |
| I | Y | Newcastle–Ottawa Scale used for observational studies; quality rated and poor-quality studies excluded; Supplementary Table 1 |
| J | N | Primary study funding sources not reported; review funding declared (Beijing Institute of Otolaryngology, NSFC) |
| K | Y | Random-effects model for regression coefficients and ORs; heterogeneity assessed with I²; subgroup and sensitivity analyses performed |
| L | PY | Sensitivity analyses conducted by omitting studies, but no formal meta-regression by RoB performed |
| M | Y | Discussion acknowledges limitations due to study quality and heterogeneity, interprets results cautiously |
| N | Y | Heterogeneity reported (I² 67–99% depending on outcome); subgroup analyses by study design, sample size, hearing assessment; heterogeneity discussed in text |
| O | Y | Funnel plots presented (Figures 4–5); publication bias discussed, particularly for OR-based meta-analyses |
| P | Y | Declared no conflicts of interest; review funding from NSFC and Beijing Institute of Otolaryngology |
| **Quality Rating:Moderate** | | |

| 6. **Yu et al., 2024** | | |
| --- | --- | --- |
| **Item** | **Rating (Y / N / PY)** | **Comments** |
| A | Y | Population: adults ≥18; Exposure: adult-onset hearing loss; Comparison: normal hearing; Outcomes: cognitive decline, MCI, dementia clearly defined |
| B | Y | Pre-registered in PROSPERO (CRD42016048835); methods predefined, no deviations reported |
| B | Y | Included only cohort studies with ≥2 years follow-up; excluded cross-sectional and intervention-only studies; rationale provided |
| D | Y | Searched PubMed, Embase, PsycINFO, Web of Science, Cochrane Library, CRD; supplementary references and grey literature checked; search strategy fully described |
| E | Y | Five authors independently screened titles, abstracts, and full texts; disagreements resolved by discussion |
| F | Y | Four authors independently extracted study data; discrepancies resolved through discussion |
| G | Y | PRISMA flowchart provided; reasons for exclusions clearly stated (n=299 assessed, 50 included) |
| H | Y | Table 1 (pages 5–6) includes sample sizes, age, sex, education, hearing assessment, hearing aid use, follow-up, and outcomes; narrative description included |
| I | Y | Used Mixed Methods Appraisal Tool (MMAT) for cohort studies; results in Supplementary Table 3; 23 studies met ≥4/5 quality criteria |
| J | N | Funding sources for primary studies not reported; review funding reported (Alzheimer’s Research UK, NIHR, UCL Hospitals BRC) |
| K | Y | Random-effects meta-analysis; separate pooling for HR, OR, RR; subgroup and meta-regression performed; I² reported |
| L | PY | Narrative discussion of study quality; sensitivity analyses conducted; no formal meta-regression by RoB performed |
| M | Y | Discussion addresses quality of studies, heterogeneity, and potential confounding; results interpreted cautiously |
| N | Y | I² reported (0–93% across analyses); heterogeneity sources discussed (age, hearing assessment method, follow-up, severity, covariates) |
| O | Y | Funnel plots inspected and Egger’s test performed (p>0.05 for most outcomes); discussed limitations in small subgroups |
| P | Y | Declared no conflicts of interest; review funding reported (ARUK, NIHR, UCL Hospitals BRC) |
| **Quality Rating:Moderate** | | |

| 7. **Liang et al., 2021** | | |
| --- | --- | --- |
| **Item** | **Rating (Y / N / PY)** | **Comments** |
| A | Y | Population: adults ≥18; Exposure: hearing loss; Comparison: normal hearing; Outcomes: dementia, Alzheimer's disease clearly defined; inclusion/exclusion criteria fully reported (pp.1–3, Table 1) |
| B | PY | Methods described in detail; no PROSPERO registration mentioned; no explicit statement about protocol deviations (pp.2–3) |
| B | Y | Only prospective cohort studies included; cross-sectional, retrospective, and intervention-only studies excluded with rationale provided (pp.2–3) |
| D | Y | Searched PubMed, Embase, Cochrane Library with combined MeSH and keywords; references and backward/forward citation checked; English-only restriction noted (pp.2–3) |
| E | Y | Two authors independently screened titles, abstracts, and full texts; disagreements resolved by consensus with corresponding author (pp.2–3) |
| F | Y | Two authors independently extracted data; cross-checked for accuracy; discrepancies resolved by discussion (pp.2–3) |
| G | Y | PRISMA flow diagram provided (Figure 1, p.3); reasons for exclusion clearly listed (e.g., retrospective studies, wrong outcome) |
| H | Y | Table 1 summarizes participant characteristics, hearing loss assessment, follow-up, dementia/AD outcomes; NOS quality scores provided (Tables 1–2, pp.4–6) |
| I | Y | Newcastle–Ottawa Scale (NOS) applied; scores from 7–9 indicate good quality (Table 2, p.5) |
| J | N | Funding sources for primary studies not reported; review funding disclosed (Fellowship of China Postdoctoral Science Foundation, NSFC, Jiangsu Provincial Key R&D) (p.13) |
| K | Y | Random-effects model applied; HRs pooled for all-cause dementia and AD; heterogeneity assessed with I² and sensitivity analyses performed (pp.7–10, Figures 2–5) |
| L | PY | Sensitivity analyses conducted by sequentially omitting studies; RoB discussed qualitatively; no formal meta-regression by NOS scores performed (pp.7–10) |
| M | Y | Discussion acknowledges study quality, heterogeneity, and potential confounders; results interpreted cautiously (pp.10–12) |
| N | Y | I² values reported (86% for dementia, 2% for AD); subgroup analyses by hearing assessment, dementia validation, follow-up, APOE adjustment conducted (Figures 3–4, pp.8–9) |
| O | Y | Funnel plots and trim-and-fill analysis for all-cause dementia; visual inspection suggested low risk for AD (Figures 6A–B, pp.11) |
| P | Y | Declared no conflicts of interest; review funding reported (p.13) |
| **Quality Rating:Moderate** | | |

| 8. **Wei et al., 2017** | | |
| --- | --- | --- |
| **Item** | **Rating (Y / N / PY)** | **Comments** |
| A | Y | Population: adults ≥55; Exposure: hearing impairment; Comparison: normal hearing; Outcomes: mild cognitive impairment (MCI) and dementia clearly defined |
| B | PY | Methods described in advance; PRISMA guideline followed; no PROSPERO registration; deviations not explicitly addressed |
| B | Y | Only prospective cohort studies included; cross-sectional and intervention-only studies excluded; rationale provided |
| D | Y | Searched PubMed, Embase, and Web of Science; additional studies from references of reviews; search strategy and keywords fully described (pp.2–3) |
| E | Y | Two reviewers independently screened titles, abstracts, and full texts; discrepancies resolved by discussion (pp.3–4) |
| F | Y | Two reviewers independently extracted study data (sample size, age, hearing assessment, cognitive outcomes, covariates); discrepancies reconciled by discussion (pp.3–4) |
| G | Y | PRISMA flow diagram provided (Figure 1, p.5); reasons for full-text exclusions listed (e.g., no hearing assessment, non-prospective cohort) |
| H | Y | Table 1 (pp.5–7) summarizes sample sizes, age, follow-up, assessment methods for hearing and cognition, prevalence, and covariates; narrative description included |
| I | Y | Newcastle–Ottawa Scale (NOS) used; total scores reported (range 5–9, mean 8.6); all studies assessed for selection, comparability, and outcome (Table 2, p.9) |
| J | N | Funding sources for primary studies not reported; review declared no funding (p.12) |
| K | Y | Random-effects meta-analysis; RR pooled for MCI and dementia; heterogeneity (I²) reported; sensitivity analyses conducted (pp.4–5, Figures 2–3) |
| L | PY | Sensitivity analyses conducted by excluding studies with extreme results; narrative discussion of quality; no meta-regression by NOS score performed (pp.8–9) |
| M | Y | Limitations due to study quality and heterogeneity discussed; interpretations cautious (pp.9–10) |
| N | Y | I² reported: MCI (0%, p=0.411), dementia (81.4%, p<0.001); heterogeneity sources discussed, including follow-up duration, hearing assessment, and cognitive outcome differences (pp.8–9) |
| O | Y | Begg’s test performed; funnel plots assessed; publication bias detected for dementia but resolved after excluding extreme studies; discussed in text (pp.8–9) |
| P | Y | Authors declared no conflicts of interest; review received no funding (p.12) |
| **Quality Rating:Moderate** | | |

| 9. **Lau et al., 2022** | | |
| --- | --- | --- |
| **Item** | **Rating (Y / N / PY)** | **Comments** |
| A | Y | Population: adults ≥50 years; Exposure: age-related hearing loss (peripheral and central); Comparison: normal hearing; Outcomes: MCI and early dementia clearly defined |
| B | Y | Protocol registered in PROSPERO (CRD42017076183); methods predefined; no major deviations reported |
| B | Y | Included cross-sectional, cohort, and longitudinal observational studies; RCTs excluded as irrelevant to observational association; rationale provided (pp.5–6) |
| D | Y | Searched MEDLINE and Cochrane Library; expert-recommended studies included; search strategy fully reported in Appendix 1 (p.6) |
| E | Y | Three authors independently screened titles, abstracts, and full texts; discrepancies resolved by consensus (p.6) |
| F | Y | Three authors independently extracted study data (population, sample size, hearing assessment, cognitive assessment, follow-up, covariates); discrepancies resolved through discussion (pp.6–7) |
| G | Y | Table 2 lists full-text exclusions with reasons (pp.30–31); PRISMA flowchart also presented (Figure 1, p.7) |
| H | Y | Table 3 (pp.32–34) provides detailed study characteristics, sample size, cognitive and hearing assessments, follow-up; narrative synthesis also included |
| I | Y | Newcastle–Ottawa Scale applied for cohort studies; all cohort studies scored ≥6/9; quality of cross-sectional studies described qualitatively (Table 4, pp.36–37) |
| J | N | Funding sources for primary studies not reported; review received no specific funding (pp.18, Financial Support section) |
| K | Y | Random-effects model used; RRs for prevalence and incidence outcomes calculated; heterogeneity assessed with I²; RevMan software used (p.6–7) |
| L | PY | Sensitivity analyses conducted by excluding outliers; qualitative discussion of study quality; no formal meta-regression based on RoB scores (pp.7, 17) |
| M | Y | Discussion acknowledges study quality, heterogeneity, and potential confounders; results interpreted cautiously (pp.16–18) |
| N | Y | I² reported for all pooled analyses; sources of heterogeneity discussed (hearing assessment method, follow-up length, study design) (pp.11–12) |
| O | PY | Funnel plots or Egger’s test not formally conducted; narrative discussion acknowledges potential publication bias due to observational nature of included studies (pp.17–18) |
| P | Y | Authors declared no conflicts of interest; no specific funding received (pp.18) |
| **Quality Rating:Moderate** | | |

# Supplementary 3:Overlap of primary studies across included meta-analyses (CCA analysis)

| **Dementia** | | | | | | |
| --- | --- | --- | --- | --- | --- | --- |
| **Study** | **Dhanda 2024** | **Loughrey 2017** | **Yu 2024** | **Liang 2021** | **Wei 2017** | **Number of occurrences** |
| Deal (2017) | 1 | 0 | 1 | 0 | 0 | 2 |
| Lin (2011) | 1 | 1 | 0 | 1 | 1 | 4 |
| Deal (2016) | 0 | 1 | 0 | 0 | 0 | 1 |
| Gallacher (2012) | 0 | 1 | 1 | 1 | 1 | 4 |
| Lin (2013) | 0 | 0 | 1 | 1 | 0 | 2 |
| Fischer (2016) | 0 | 0 | 1 | 1 | 0 | 2 |
| Alattar (2019) | 0 | 0 | 1 | 0 | 0 | 1 |
| Su (2017) | 0 | 0 | 1 | 1 | 0 | 2 |
| Yu and Woo (2019) | 0 | 0 | 1 | 1 | 0 | 2 |
| Deal (2017) | 0 | 0 | 0 | 1 | 0 | 1 |
| Gao (2020) | 0 | 0 | 0 | 1 | 0 | 1 |
| Chen and Zhou (2020) | 0 | 0 | 0 | 1 | 0 | 1 |
| Gates (2002) | 0 | 0 | 0 | 0 | 1 | 1 |
| Gates (2011) | 0 | 0 | 0 | 0 | 1 | 1 |
| Deal (2017) | 0 | 0 | 0 | 0 | 1 | 1 |
| Gurgel (2014) | 0 | 0 | 0 | 0 | 1 | 1 |
| Golub (2017) | 0 | 0 | 0 | 0 | 1 | 1 |
| Number of meta-analyses (R):5 Number of original studies (N):17  Number of study combinations (Nr):28 | | | | | | |
| CCA calculation formula:(Nr - N) / (R * N - N)  CCA value:0.161764705882353 Duplication Rate (%):16.1764705882353% | | | | | | |

| **Alzheimer’s disease** | | | | |
| --- | --- | --- | --- | --- |
| **Study** | **Loughrey 2017** | **Yu 2024** | **Liang 2021** | **Number of occurrences** |
| Gallacher (2012) | 1 | 0 | 0 | 1 |
| Lin et al (2011) | 1 | 0 | 1 | 2 |
| Deal (2017) | 0 | 1 | 1 | 2 |
| Lin (2013) | 0 | 1 | 1 | 2 |
| Su (2017) | 0 | 1 | 1 | 2 |
| Number of meta-analyses (R):3 Number of original studies (N):5  Number of study combinations (Nr):9 | | | | |
| CCA calculation formula:(Nr - N) / (R * N - N)  CCA value:0.4 Duplication Rate (%):40% | | | | |

| **Cognitive impairment** | | | | | | | |
| --- | --- | --- | --- | --- | --- | --- | --- |
| **Study** | **Dhanda 2024** | **Loughrey 2017** | **Fu 2022** | **Yu 2024** | **Wei 2017** | **Lau 2022** | **Number of occurrences** |
| Alattar (2019) | 1 | 0 | 0 | 1 | 0 | 0 | 2 |
| Fischer (2017) | 1 | 0 | 0 | 1 | 0 | 1 | 3 |
| Lin (2013) | 1 | 1 | 0 | 1 | 1 | 1 | 5 |
| Gallacher (2012) | 0 | 1 | 0 | 1 | 1 | 1 | 4 |
| Kiely (2012) | 0 | 0 | 0 | 0 | 0 | 0 | 0 |
| Dai (2021) | 0 | 0 | 1 | 0 | 0 | 0 | 1 |
| Hung (2015) | 0 | 0 | 1 | 0 | 0 | 0 | 1 |
| Wang (2021) | 0 | 0 | 1 | 0 | 0 | 0 | 1 |
| Wang (2020) | 0 | 0 | 1 | 0 | 0 | 0 | 1 |
| Luo (2018) | 0 | 0 | 1 | 0 | 0 | 0 | 1 |
| Li (2017) | 0 | 0 | 1 | 0 | 0 | 0 | 1 |
| Chen and Zhou (2022) | 0 | 0 | 1 | 0 | 0 | 0 | 1 |
| Pan (2021) | 0 | 0 | 1 | 0 | 0 | 0 | 1 |
| Guo (2020) | 0 | 0 | 1 | 0 | 0 | 0 | 1 |
| Mukadam (2019) | 0 | 0 | 1 | 0 | 0 | 0 | 1 |
| Han (2019) | 0 | 0 | 1 | 0 | 0 | 0 | 1 |
| Gao (2020) | 0 | 0 | 1 | 0 | 0 | 0 | 1 |
| Chen and Zhou (2020) | 0 | 0 | 1 | 0 | 0 | 0 | 1 |
| Chen (2021) | 0 | 0 | 1 | 0 | 0 | 0 | 1 |
| Chen and Lu (2019) | 0 | 0 | 1 | 0 | 0 | 0 | 1 |
| Yu and Woo (2019) | 0 | 0 | 1 | 0 | 0 | 0 | 1 |
| Ma (2020) | 0 | 0 | 1 | 0 | 0 | 0 | 1 |
| Su (2017) | 0 | 0 | 1 | 0 | 0 | 1 | 2 |
| Deal (2017) | 0 | 0 | 0 | 1 | 0 | 1 | 2 |
| Fischer (2016) | 0 | 0 | 0 | 0 | 1 | 0 | 1 |
| Heywood (2017) | 0 | 0 | 0 | 0 | 0 | 1 | 1 |
| Number of meta-analyses (R):6 Number of original studies (N):26  Number of study combinations (Nr):37 | | | | | | | |
| CCA calculation formula:(Nr - N) / (R * N - N)  CCA value:0.0846153846153846 Duplication Rate (%):8.46153846153846% | | | | | | | |

# Supplementary 4: Sensitivity analysis

Sensitivity analysis for association between hearing loss and cognitive impairment in overall adults (A) and older adults (B)

Sensitivity analysis for dementia (C)

# Supplementary 5: GRADE assessment and CLASS of evidence for the association between hearing loss and cognitive outcomes

| **Outcome** | **Research** | **GRADE** | | | | | | | | **Class of Evidence** |
| --- | --- | --- | --- | --- | --- | --- | --- | --- | --- | --- |
|  |  | **Risk of bias** | **Inconsistency** | **Indirectness** | **Imprecision** | **Publication Bias** | **Upgrade** | **Certainty** | **Note** |  |
| **Cognitive impairment** | Dhanda 2024 | Not serious | Not serious | Not serious | Not serious | Undetected | 0 | Moderate | CI narrow,  I² moderate | Class II-Highly suggestive |
|  | Loughrey 2017 | Not serious | Not serious | Not serious | Not serious | Undetected | 0 | High | I²=0%, CI narrow |  |
|  | Fu 2022 | Not serious | Serious | Not serious | Not serious | Undetected | 1 (large effect) | Moderate | High heterogeneity, OR≈2 → upgrade 1 level |  |
|  | Yu 2024 | Not serious | Not serious | Not serious | Not serious | Undetected | 0 | High | CI narrow, I²=0% |  |
|  | Wei 2017 | Not serious | Not serious | Not serious | Not serious | Undetected | 0 | High | CI narrow, I²=0% |  |
|  | Lau 2022 | Not serious | Not serious | Not serious | Not serious | Undetected | 0 | High | CI narrow, I²=0% |  |
| **Cognitive impairment-the elderly** | Loughrey 2017 | Not serious | Not serious | Not serious | Not serious | Undetected | 0 | High | I²=0%, CI narrow | Class II-Highly suggestive |
|  | Fu 2022 | Not serious | Serious | Not serious | Not serious | Undetected | 1 (large effect) | Moderate | High heterogeneity, OR≈2 → upgrade 1 level |  |
|  | Yu 2024 | Not serious | Not serious | Not serious | Not serious | Undetected | 0 | High | CI narrow, I²=0% |  |
|  | Wei 2017 | Not serious | Not serious | Not serious | Not serious | Undetected | 0 | High | CI narrow, I²=0% |  |
|  | Lau 2022 | Not serious | Not serious | Not serious | Not serious | Undetected | 0 | High | CI narrow, I²=0% |  |
| **Dementia** | Dhanda 2024 | Not serious | Serious | Not serious | Not serious | Undetected | 0 | Moderate | Moderate heterogeneity, narrow CI | Class II-Highly suggestive |
|  | Loughrey 2017 | Not serious | Serious | Not serious | Not serious | Undetected | 0 | Moderate | High heterogeneity, consistent direction |  |
|  | Yu 2024 | Not serious | Serious | Not serious | Not serious | Undetected | 0 | Low | High heterogeneity, no upgrade factor |  |
|  | Liang 2021 | Not serious | Serious | Not serious | Not serious | Undetected | 0 | Low | Very high heterogeneity |  |
|  | Wei 2017 | Not serious | Serious | Not serious | Not serious | Undetected | 1 (large effect) | Moderate | High heterogeneity, RR>2 triggers 1-level upgrade |  |
| **Dementia-the elderly** | Loughrey 2017 | Not serious | Serious | Not serious | Not serious | Undetected | 0 | Moderate | High heterogeneity, consistent direction | Class II-Highly suggestive |
|  | Yu 2024 | Not serious | Serious | Not serious | Not serious | Undetected | 0 | Low | High heterogeneity, no upgrade factor |  |
|  | Liang 2021 | Not serious | Serious | Not serious | Not serious | Undetected | 0 | Low | Very high heterogeneity |  |
|  | Wei 2017 | Not serious | Serious | Not serious | Not serious | Undetected | 1 (large effect) | Moderate | High heterogeneity, RR>2 triggers 1-level upgrade |  |
| **Alzheimer’s disease--the elderly** | Loughrey 2017 | Not serious | Serious | Not serious | Serious | Undetected | 0 | Low | Only 2 studies, wide CI, no significant association | Class I-Convincing |
|  | Yu 2024 | Not serious | Not serious | Not serious | Not serious | Undetected | 0 | Moderate | CI narrow, I²=0%, sample large |  |
|  | Liang 2021 | Not serious | Not serious | Not serious | Not serious | Undetected | 1 (large effect) | Moderate | OR>2, I² low, direction consistent |  |
| **Cognitive function outcomes** | Satheesan 2025 Overall cognition function | Not serious | Not serious | Not serious | Not serious | Undetected | 0 | Moderate | I² = 14%, CI narrow | / |
|  | Satheesan 2025 Memory | Not serious | Not serious | Not serious | Not serious | Undetected | 0 | High | I² = 0%, CI narrow |  |
|  | Satheesan 2025 Executive function | Not serious | Serious | Not serious | Not serious | Undetected | 0 | Low | I² = 67%, moderate heterogeneity |  |
|  | Taljaard 2016 General cognition | Not serious | Serious | Not serious | Not serious | Undetected | 0 | Moderate | I² = 51.78% |  |
|  | Taljaard 2016 Attention / Processing speed | Not serious | Serious | Not serious | Not serious | Undetected | 1 (large effect) | Moderate | SMD ≥ 0.5, I² = 53.67% |  |
|  | Taljaard 2016 Working memory | Not serious | Serious | Not serious | Not serious | Undetected | 0 | Low | I² = 61.18% |  |
|  | Taljaard 2016 Long-term memory | Not serious | Serious | Not serious | Not serious | Undetected | 0 | Low | I² = 72.51% |  |
|  | Taljaard 2016 Executive function | Not serious | Not serious | Not serious | Not serious | Undetected | 0 | High | I² = 4.29%, CI narrow |  |
|  | Taljaard 2016 Language ability | Not serious | Not serious | Not serious | Not serious | Undetected | 0 | High | I² = 0%, CI narrow |  |
